# Estimating distributions of health state severity for the global burden of disease study

**DOI:** 10.1186/s12963-015-0064-y

**Published:** 2015-11-18

**Authors:** Roy Burstein, Tom Fleming, Juanita Haagsma, Joshua A. Salomon, Theo Vos, Christopher JL. Murray

**Affiliations:** Department of Global Health, Institute for Health Metrics and Evaluation, University of Washington, 2301 5th Avenue #600, Seattle, WA 98121 USA; Department of Public Health, Erasmus Medical Center, Erasmus University Rotterdam, Rotterdam, The Netherlands; Department of Global Health and Population, Harvard T.H. Chan School of Public Health, Boston, MA USA

**Keywords:** Global burden of disease, Non-fatal outcomes, Medical expenditures panel survey (MEPS), Functional health status, Disability weights, 12-item short form health survey (SF-12)

## Abstract

**Background:**

Many major causes of disability in the Global Burden of Disease (GBD) study present with a range of severity, and for most causes finding population distributions of severity can be difficult due to issues of sparse data, inconsistent measurement, and need to account for comorbidities. We developed an indirect approach to obtain severity distributions empirically from survey data.

**Methods:**

Individual-level data were used from three large population surveys from the US and Australia that included self-reported prevalence of major diseases and injuries as well as generic health status assessments using the 12-Item Short Form Health Survey (SF-12). We developed a mapping function from SF-12 scores to GBD disability weights. Mapped scores for each individual respondent were regressed against the reported diseases and injuries using a mixed-effects model with a logit-transformed response variable. The regression outputs were used to predict comorbidity-corrected health-state weights for the group of individuals with each condition. The distribution of these comorbidity-corrected weights were used to estimate the fraction of individuals with each condition falling into different GBD severity categories, including asymptomatic (implying disability weight of zero).

**Results:**

After correcting for comorbid conditions, all causes analyzed had some proportion of the population in the asymptomatic category. For less severe conditions, such as alopecia areata, we estimated that 44.1 % [95 % CI: 38.7 %-49.4 %] were asymptomatic while 28.3 % [26.8 %-29.6 %] of anxiety disorders had asymptomatic cases. For 152 conditions, full distributions of severity were estimated. For anxiety disorders for example, we estimated the mean population proportions in the mild, moderate, and severe states to be 40.9 %, 18.5 %, and 12.3 % respectively. Thirty-seven of the analyzed conditions were used in the GBD 2013 estimates and are reported here.

**Conclusion:**

There is large heterogeneity in the disabling severity of conditions among individuals. The GBD 2013 approach allows explicit accounting for this heterogeneity in GBD estimates. Existing survey data that have collected health status together with information on the presence of a series of comorbid conditions can be used to fill critical gaps in the information on condition severity while correcting for effects of comorbidity. Our ability to make these estimates may be limited by lack of geographic variation in the data and by the current methodology for disability weights, which implies that severity must be binned rather than expressed in as a full distribution. Future country-specific data collection efforts will be needed to advance this research.

**Electronic supplementary material:**

The online version of this article (doi:10.1186/s12963-015-0064-y) contains supplementary material, which is available to authorized users.

## Background

Disability weights are a critical component in estimating the burden of non-fatal disease, allowing for comparison of time lived with different conditions in order to quantify years lived with disability (YLDs) [1], and ultimately for comparability with years of life lost (YLL) to create the summary composite disability-adjusted life year (DALY) [2], as well as the health-adjusted life expectancy [3] summary measure. Disability weights are measured on a zero to one scale where one is a health state loss that is equivalent to death and zero represents no functional limitation. The disability weight is meant to capture the severity of functional limitations in different domains of health, but not the welfare or social welfare loss associated with a given health state [4, 5].

For the Global Burden of Disease, Injuries, and Risk Factors (GBD) 2010 study, disability weights were measured through general population surveys in five countries (United States, Peru, Bangladesh, Indonesia, and Tanzania) as well as through an open internet survey with participation from 167 countries. The main mode of measurement used in these surveys was a simple paired comparison question in which respondents considered two outcomes described briefly in lay language, and decided which outcome they regarded as the more healthy of the pair. Short descriptions were used so that respondents of varying degrees of educational attainment could comprehend them and make a judgment on the level of health associated with different states. In 2013, the same methods were applied in web-based sample surveys among representative population samples from Hungary, Italy, the Netherlands, and Sweden. For GBD 2013 the data from the GBD 2010 Disability Weights Measurement study and European Disability Weights Measurement study were combined, resulting in a set of disability weights based on the valuations of 60,890 people [4, 6, 7].

For some conditions, the loss of function described in the short description captures the typical case of a condition. For example, the health state description for an amputated toe is straightforward: “has lost one toe, leaving occasional pain and tingling in the stump”. For many conditions, however, there is a spectrum of severity. The severity spectrum for a number of conditions such as chronic obstructive pulmonary disease (COPD), heart failure, or anxiety disorders was considered in the design of the study by developing lay descriptions and measuring associated disability weights for more than one level of severity. To the extent possible, these were based on standard clinical classification systems.

Empirical measurement of the variation of severity across individuals using published or unpublished data is challenging for five main reasons. First, many published studies on severity distributions use clinical or biometric criteria and not functional health status measurements. For example, the New York Heart Association classification of heart failure is widely used and, while symptom-based, is not directly linked to a functional health status instrument [8]. The same applies to the classification of major depression and anxiety disorders in ICD-10 or the Diagnostic and statistical manual of mental disorders fourth edition (DSM-IV) which are based on symptom counts. Second, where functional health status data have been collected, many different instruments have been used such as EQ-5D [9], the 12-item Short Form (SF-12) [10], the Health Utilities Index [11], the Assessment of Quality of Life [12] or a multitude of other disease-specific quality of life and functioning instruments. Mapping between these various instruments and the GBD disability weights requires an extra empirical step for which there may be only limited data [13]. Third, well-characterized [14–16] problems of interpersonal incomparability known as differential item functioning across individuals in functional health status instruments can complicate the assessment of severity distributions. Differential item functioning occurs when respondents from different groups but living in a similar health state will give different responses to questionnaires. The presence of differential item functioning tends to increase the variance of measured functional health status in a sample and lead to an increase in the number of individuals reporting severe or no disability for a given condition. Differential item functioning is likely to be a greater challenge in this regard with samples that vary in educational attainment, linguistic, or cultural background. Fourth, in GBD 2010 disability weights pertained to individuals in health states due to one condition at a time, and therefore empirical severity distributions from surveys need to be corrected for comorbidities. Identifying the marginal severity distribution due to a condition from measured data requires the use of some form of statistical model. An alternative would be to only consider individuals who have a condition of interest without any comorbidity. With a large number of health states considered in GBD this would exclude the majority of respondents and potentially lead to considerable selection bias, and would almost certainly bias observed severity downward. The need to estimate the marginal severity distribution limits the use of many published studies on severity that do not take comorbidity into account, particularly at older ages when comorbidity is the norm rather than the exception [17, 18]. Finally, data from clinical cases may be biased because they likely reflect more severe cases which sought medical attention.

In this paper, we describe the analysis of three large population sample datasets to supplement information available in other studies on the distribution of severity associated for several conditions in the GBD. The results here are specific to the GBD 2013 round of estimates. Conditions were selected if 1) there were little or no credible data on severity from systematic reviews of the published and unpublished literature, or 2) data on severity were not easily comparable with GBD health state descriptions.

## Methods

We have taken advantage of three large available datasets to estimate severity distributions: the US Medical Expenditure Panel Survey (MEPS) [19], the National Epidemiologic Survey on Alcohol and Related Conditions (NESARC) [20], and the 1997 Australian National Survey of Mental Health and Wellbeing of Adults (NSMHWB) [21]. MEPS and NESARC have multiple measurements for the same individual. All three collected functional health status information and provide information on a broad range of comorbidities. After describing these datasets in detail, we describe the analysis in three stages: mapping from SF-12 to GBD disability weight space, development of a statistical model for individual functional health status responses, and estimation of severity distributions.

### Data

The three national surveys collected information using SF-12 and information on a range of comorbid conditions. The SF-12 questionnaire is a widely used measure of generic health status. SF-12 is summarized into the physical and mental component scores (PCS-12 and MCS-12 respectively), which are standardized to a mean of 50 and a standard deviation of 10, such that a higher score represents better physical and emotional functioning. Each summary score corresponds to a four-week recall period. As its name suggests, the SF-12 asks only 12 questions about functioning but has been shown to be comparable with the longer 36-item short form survey [22].

MEPS is a large-scale overlapping continuous panel survey of the non-institutionalized US population whose primary purpose is to collect information on the use and cost of healthcare. Panels are two years long and are conducted in five rounds, with data collection rounds every four to six months. A new panel begins every year, while the previous panel is in its second year [23]. Each panel typically contains about 30,000 to 35,000 individual respondents. MEPS was initiated in 1996, but only began collecting SF-12 responses in 2000, and thus we only used data from 2000–2010. Data from these years were pooled for this analysis. Respondents self-administer the SF-12 twice per panel, at rounds two and four, typically eight to 12 months apart. Only adults 18 years and older responded to the SF-12. Of these, we were able to use MCS-12 and PCS-12 scores for 203,960 measurements, taken from 119,676 individuals.

Medical conditions are recorded in MEPS for one of three reasons: 1) they were reported as a reason for a medical event, i.e. a health service contact in primary care or as an inpatient or at the purchase of a drug, 2) the condition was reported as the reason for one or more disability days, or 3) the condition was “bothering” the person during the reference period. The first of these options is by far the most common source of diagnostic information. Conditions were recorded as verbatim text and coded to ICD-9CM three digit codes by professional medical coders. Error rates per coder are not expected to exceed 2.5 % [24]. These codes were not validated with medical providers, though they have been shown to have high sensitivity. A sensitivity study also found that rates will improve as condition categories are aggregated [25]. We mapped ICD-9 codes to 152 categories which were consistent with the GBD cause list (see mapping in Additional file 6), this aggregation likely improved the sensitivity further.

NESARC was conducted in two waves, the first occurring in 2001–2002 and the second in 2004–2005. NESARC is a representative sample of the non-institutionalized US population aged 18 and older. NSMHWB offers a representative sample of adults living in private dwellings in Australia and was conducted in 1997. Respondents to both surveys were administered the SF-12.

In NESARC and NSMHWB, conditions were measured differently than in MEPS. Respondents to the NSMHWB were diagnosed for mental and substance use disorders via the Composite International Diagnostic Interview (CIDI), a standard questionnaire form based on criteria of ICD-10 and DSM-IV [21]. Most mental conditions from NESARC were diagnosed using an operationalized set of questions from the DSM-IV using the Alcohol Use Disorder and Associated Disabilities Interview Schedule-IV, or AUDADIS-IV [20]. MEPS did not use DSM-IV and relied on self-report of both mental and physical conditions. NESARC offered 12-month prevalence of diagnoses and NSMHWB offered both one month and 12 month diagnoses. We explore the sensitivity of disability measurements to different diagnostic periods later in this paper. Health measurements for 10,641 NSMHWB respondents were used, and 75,656 measurements from 42,494 respondents from NESARC were used. By design, NESARC and NSMHWB had collected information on significantly fewer physical conditions than MEPS. We had information on 26 physical and mental conditions from NESARC and 17 conditions from NSMHWB. Correction for comorbidities in these surveys was thus necessarily less comprehensive.

NESARC offered the benefit of splitting up drug dependence categories while still retaining a large enough sample in each category. This was particularly useful for estimating severities in the GBD cocaine, cannabis, and amphetamine dependence categories [26] (Table 1).

**Table 1:** Number of respondents by age, sex, and dataset to be included in the analysis. Each MEPS and NESARC respondent averaged 1.9 observations. AHS respondents only had one observation

	MEPS		NESARC		NSMHWB	
age group	males	females	males	females	males	females
18 ‐ 19	2,466	2,488	187	138	120	148
20 ‐ 24	5,028	5,578	1,512	1,587	341	460
25‐ 29	4,884	5,765	1,425	2,011	438	610
30 ‐ 34	5,174	6,072	1,637	2,272	502	639
35 ‐ 39	5,456	6,339	1,856	2,499	578	733
40 ‐ 44	5,631	6,487	2,040	2,668	553	616
45 ‐ 49	5,432	6,393	1,941	2,202	456	528
50 ‐54	4,985	5,793	1,758	2,037	359	489
55 ‐ 59	4,215	4,904	1,417	1,882	351	336
60 ‐ 64	3,290	3,847	1,165	1,481	276	316
65 ‐ 69	2,612	3,097	974	1,286	285	293
70 ‐ 74	2,018	2,654	894	1,323	201	299
75 ‐ 79^a^	1,725	2,350	754	1,274	245	469
80 +	1,784	3,209	953	1,907		
	54,700	64,976	18,513	24,567	4,705	5,936

^a^75‐79 age group is 75+ for NSMHWB

### Mapping SF-12 to disability weights

To make use of the extensive data collected using SF-12 in MEPS, NESARC and NSMHWB for assessing the distribution of severity, individual SF-12 results had to be mapped to an equivalent disability weight. SF-12 produces two summary scores, the MCS and the PCS as noted. To develop a mapping we selected 62 of the 234 lay descriptions used in the GBD 2010 disability weight study that represented the full range of disability weight values covering the spectrum from most mild (mild distance vision impairment: “has some difficulty with distance vision, for example reading signs, but no other problems with eyesight”, with an associated disability weight of 0.004) to most severe (active phase of schizophrenia: “hears and sees things that are not real and is afraid, confused, and sometimes violent. The person has great difficulty with communication and daily activities, and sometimes wants to harm or kill himself (or herself)”, with an associated disability weight of 0.763). We used a convenience sample of respondents to complete the SF-12 form for the hypothetical individual living in the state described in each of the 62 conditions; respondents were not asked to complete the SF-12 for themselves but for an individual with the health state described in the lay description. These samples were done at IHME offices in Seattle and at two GBD training workshops in Greece. Each respondent completed SF-12 responses for up to 50 randomly selected states, in random order, out of the 62. A total of 3,791 responses were collected.

Disability weights are associated with both mental and physical disability. To examine the relative contributions of each, we first regressed the GBD disability weight for each of the states on the MCS and PCS scores. The coefficients in this regression were −0.0072 and −0.0045, respectively. However, examination of the results showed that the states for severe depression and acute state schizophrenia had only severe MCS limitations which were driving these coefficients. Exclusion of these two states showed that the coefficients were −0.0055 and −0.0056 for MCS and PCS respectively. The nearly equal coefficients imply that MCS and PCS scores contribute about equally to the disability weights. To simplify the mapping of continuous MCS and PCS scores into the disability weight space needed for this analysis, we combined the MCS and PCS scores into an overall score through simple addition as is commonly done with SF-12.

**Fig. 1:**
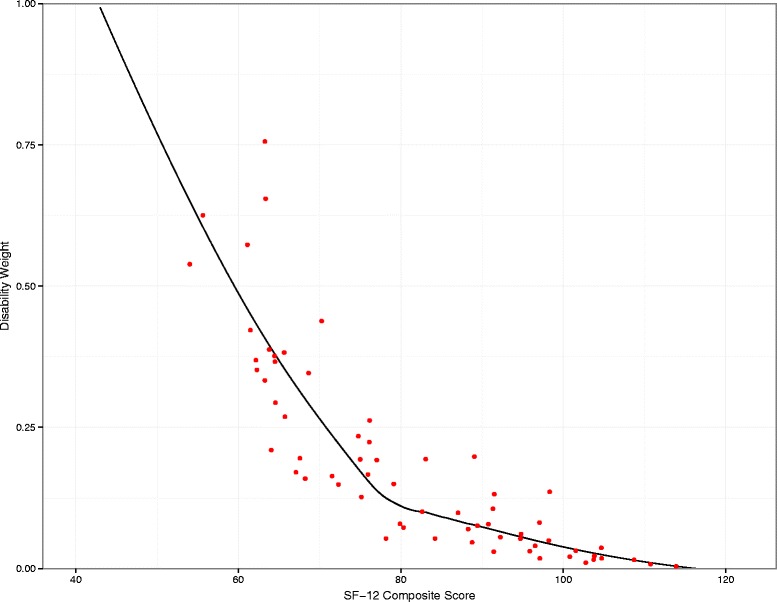
Disability weight mapping as regressed on estimated SF-12 scores from survey. Each dot represents a mean Health State weight

Given some outliers in the responses, we chose to use the trimmed mean score for each lay description group, first by excluding all responses that were more than two median absolute deviations (MAD) from the median within each lay description group. 650 observations (19 %) were dropped in this step, 53.8 % from the low end and 46.2 % from the high end. After correcting for outliers, the simple rank order correlation mean DW and mean SF-12 was −0.706. The relationship was not linear. To generate a smooth mapping from SF-12 combined scores to the GBD disability weight space, we used loess regression on the trimmed mean SF-12 score for each health state. Loess fits simple models to localized subsets of the data in order to explain the variation point by point, and thus allows us to define a function that is not restricted by a pre-defined form. Because disability weights are defined in the range from zero to one, we truncated the derived function at a combined SF-12 score of 116.34 (any combined score above this level was set to 0) and truncated the function at 43.0 so that any combined score less than that value was set to 1. This truncation affected 6.0 % of the observations in the population survey data described in the following subsection. See Additional file 1 for a list of lay descriptions and their associated disability weights and mean SF-12 composite scores. The final function is visualized in Fig. 1.

### Modeling individual functional health status

Using the function described above, we transformed SF-12 values from the population survey data into equivalent disability weight values. These mapped disability weights represented each respondent’s total, or cumulative, disability. To compare with condition-specific GBD disability weights in isolation in order to develop marginal severity distributions, we modeled total disability as a composition of individual conditions. Ultimately this model allowed us to determine the distribution of condition-specific weights for the populations surveyed.

For internal consistency, we assumed the same multiplicative form used for the GBD computation of comorbidity corrections; [1] the cumulative individual disability weight is a multiplicative function of the disability weights *DW*_*c*_ for all conditions *c* = 1, 2, …, *N* affecting an individual, such that:

1 $$ cumulative\kern0.5em DW\kern0.5em =\kern0.5em 1\kern0.5em -\kern0.5em {\varPi}_{c\kern0.5em =\kern0.5em 1}^N\kern0.5em \left(1\kern0.5em -\kern0.5em D{W}_c\right) $$

This multiplicative function is useful because it does not allow for an individual cumulative weight outside the bounds of zero and one, and thus the marginal disabling effect of each condition on the individual total reduces with each additional condition. A simple algebraic rearrangement of this formula allows one to retrieve the condition specific disability weight for each individual-condition combination.

We modeled the comorbidity-disability relationship using a mixed-effects model with a logit-transformed dependent variable. Binary indicator variables were assigned to each condition, and attributed to individuals’ health measurements if the medical event coincided with the time the measurement was taken. In MEPS, the condition list refers to the two rounds preceding the SF-12 response. Logit-transforming the outcome variable offers the benefit of limiting the range of the outcome disability weight between zero and one. Furthermore, a logit-transformed dependent variable defines a multiplicative relationship between the independent parameters, which is consistent with the multiplicative model for combining disability weights for YLD estimation described above. Disability weights were modeled, for each *m* measure of each *i* individual over *N* total conditions in each survey, linearly in logit-space, as follows:

2 $$ \log \kern0.5em \left(\frac{cumulative\kern0.5em D{W}_{i,m}}{1\kern0.5em -\kern0.5em  cumulative\kern0.5em D{W}_{i,m}}\right)\kern0.5em =\kern0.5em {\beta}_0\kern0.5em +\kern0.5em {\beta}_1 Condition{1}_{i,m}\kern0.5em +\kern0.5em \cdot \cdot \cdot \kern0.5em +\kern0.5em {\beta}_N Condition\kern0.5em {N}_{i.m}\kern0.5em +\kern0.5em {U}_i\kern0.5em +\kern0.5em {\varepsilon}_{i,m} $$

where Condition *j*_*im*_ (*j* = 1, 2, …, *N*) is a dummy variable indicating whether measurement *m* in individual *i*, has that condition present, and the *U*_*i*_ term is a random intercept on individual, to account for individual variations over multiple individual-measures. The model uses the only the composition of conditions within each individual to explain cumulative disability. We chose not to include age and sex in the model because we were interested in the direct effects of conditions alone on health status, and not in controlling for demographic variables that precede conditions on the causal pathway of interest, since the allocation of conditions is so dependent on age and sex. Furthermore, a simpler model without age and sex allowed us to assess how much of the observed age pattern of functional health status is accounted for by a simple comorbidity model in the validation step. The model was run separately for each survey.

Next, we estimated the effect of each condition in isolation among the population with that condition. We call this the condition-specific disability. In order to do this we re-wrote equation 1 such that we could solve for the condition specific weight of each individual with said condition:

3 $$ D{W}_{c,i,m}\kern0.5em =\kern0.5em 1\kern0.5em -\kern0.5em \frac{1\kern0.5em -\kern0.5em  cumulative\kern0.5em D{\widehat{W}}_{i,m}}{1\kern0.5em -\kern0.5em  counter\kern0.5em  factual\kern0.5em D{\widehat{W}}_{i,m}}, $$

where *cumulative DŴ*_*i*,*m*_ is the expected value of each individual measure of total disability as predicted by the model for each observation. The term *counter factual DŴ*_*i*,*m*_ represents the estimated total disability for each observation estimated using only the main effects and excluding the condition of interest. For example, if we were analyzing COPD, we could estimate the predicted disability weight for each individual recorded as having COPD excluding the effect of COPD. This produces an expected disability for these individuals taking into account all of their respective comorbidities but not the condition of interest (the counterfactual). We then removed the counterfactual disability from the predicted cumulative disability to determine the marginal effect of the condition of interest for that individual. In other words, we estimated the condition-specific disability for each individual-condition combination as the predicted cumulative individual weight portioning out the effects of all comorbid conditions.

For this analysis, we did not include the individually estimated random effects when predicting the counterfactual. The reason is that many chronic conditions are present at both waves of the data collection, and the random effect incorporates information on the distribution of severity already, as well as other individual-level variation perhaps due to differential item functioning. If we were to include the random effect in the estimation of the counterfactual we could be underestimating severity for long-term chronic conditions.

Evaluating equation 3 for each individual, we then took the mean of condition specific disability weights over the subset of the population that had the condition to determine the population mean condition-specific disability. Uncertainty in these estimates was estimated using bootstrapping: the process was done 1,000 times for each condition, re-sampling with replacements each time.

### Validation

For a simple validation, we were interested in seeing if this approach to predicting average weights would generally predict the level of total disability at the population level. We used the simple multiplicative model (equation 1) to back-estimate cumulative individual disability using the estimated condition-specific weights for each individual, accounting for individual composition of comorbidities and the model intercept. We included the intercept to account for the unmeasured disability from short-term or other conditions that were not included in the model. Disability weights that were estimated below zero were necessarily truncated to zero, as the multiplicative equation can only handle disability weight values between zero and one. We compared these estimates to the SF-12 transformed average cumulative individual disability weights for each five year age group.

### Estimating marginal severity distributions

As described above, the model allows us to easily estimate a distribution of condition-specific weights for each survey using a simple multiplicative formula for individual comorbidity. We followed these same steps to estimate distributions of health state-specific severity distributions, but instead of using the predicted cumulative disability in the numerator, we used the observed cumulative disability. It was important to use the observed cumulative disability because we wanted our estimates of health state-specific disability to reflect the observed heterogeneity in functional health seen in these surveys, and not just the distribution produced by different numbers of comorbidities, as predicted by the model. We thus distinguish the term condition-specific disability (as estimated by the model) from health-state specific disability (as estimated using the observed data). Health-state specific disability for each observation was thus estimated following a slight alteration to equation 3:

4 $$ Health\kern0.5em  state\kern0.5em D{W}_{i,m}\kern0.5em =\kern0.5em 1\kern0.5em -\kern0.5em \frac{1\kern0.5em -\kern0.5em  cumulative\kern0.5em D{W}_{i,m}}{1\kern0.5em -\kern0.5em  counter\kern0.5em  factual\kern0.5em D{\widehat{W}}_{i,m}} $$

This again gave us a distribution of condition-associated health-state specific weights amongst the population with the condition in question. To make this distribution fit with the GBD framework, we then binned the population into categories of severity for which disability weights were defined already by the GBD DW study. We set cutoffs for the bins at the midpoint between the DW values for each state. For example, if anxiety cases can be binned as asymptomatic, mild, moderate, or severe, then anything below zero would be considered asymptomatic, and the cutoffs for the mild bin would be between zero and the midpoint of the mild and moderate disability weights; the cutoffs for moderate cases would be between the mild/moderate midpoint and the moderate/severe midpoint; severe cases would be considered anything higher than the moderate/severe midpoint. See Fig. 3 for an illustration of this binning using the population with anxiety disorders from MEPS as an example.

Zero arises naturally as the upper cutoff for the asymptomatic category. Cases were considered asymptomatic for the condition of interest if the predicted counterfactual weight exceeded the observed individual cumulative weight. This results in a health state valued at a number lower than zero. For example, consider the following fictional example: an individual has anxiety, depression, and acne, with an observed SF-12 transformed disability weight of 0.13, but the model predicts the total disability of their comorbid depression and anxiety conditions alone to be 0.15. This person’s estimated acne-associated health state will be −0.02 and would thus be assumed asymptomatic for acne as we conceptualize their disability to come the combination of their comorbid conditions and not acne. The same person would not be asymptomatic for depression if for instance their counterfactual depression weight (for acne and anxiety this time) is only 0.05; then 0.08 disability will be assigned to the depressive health state. All conditions have the opportunity to ‘claim’ the comorbidity-corrected residual disability, but the amount they can ‘claim’ depends on the amount of disability estimated to be attributable to their comorbid conditions.

This analysis was run separately for each survey, and also separately for the one- and 12-month diagnoses available in NSMHWB. The two NSMHWB survey waves allowed us to compare the sensitivity of these results to diagnostic periods.

Uncertainty in distribution estimates were based off 1,000 bootstrapped datasets. As disability weights also have measured uncertainty, the 1,000 distributions produced for each condition were binned for each of the 1,000 draws of GBD disability weights used to make cutoffs. In this way, we were able to incorporate both sources of uncertainty.

### Role of funding source

Funding was provided by the Bill & Melinda Gates Foundation. The funder had no role in writing the manuscript or the decision to submit for publication.

## Results

### Model of functional health status

The results of the model represent mean condition-specific disability, and while important because they inform the counterfactual disability estimates, are not themselves used to calculate YLDs. Full model results summaries for each survey are found in Additional files 2 and 3.

In general, estimated condition-specific weights ordered themselves in a manner quite consistent with expectations. Serious cancers, mental disorders, and serious injuries had the largest estimated effects, indicating the greatest health loss, while more common and less serious conditions such as acne, benign prostatic hypertrophy, and attention-deficit hyperactivity disorder were toward the bottom of the list. Where comparisons could be made across surveys, results were somewhat mixed. For example, all three surveys placed unipolar major depressive disorder at about 0.08, while MEPS, NSMHWB, and NESARC placed anxiety at 0.05, 0.06, and 0.03 respectively. Other conditions had even larger ranges, for example MEPS estimated the mean effect of cirrhosis at 0.08, while NESARC estimated it as its largest effect, at 0.20.

Uncertainty intervals in some estimates were large, and crossed zero for 46 out of the 152 conditions classified in the MEPS dataset. Many of these were likely due to small sample sizes, especially in rarer cancers such as mouth cancer (mean DW: −0.03 [95 % CI: −0.10 to 0.04], n = 34) and testicular cancer (0 · 02 [95 % CI: −0.02 to 0.11], n = 42). Estimates for conditions we would consider similarly disabling a priori, but which had larger sample sizes, were more stable and realistic. For example, consider lung cancer (0.14 [95 %CI: 0.10 to 0.18], *N* = 439) and prostate cancer (0 · 03 [95 % CI: 0.02 to 0.04], *n* = 1057). Any measurements that fall below zero would indicate that individuals with those conditions, on average, were healthier than the mean healthy population, as represented by the model intercept. Conditions with the highest estimated mean effects from MEPS were pancreatic cancer (0.29 [95 % CI: 0.10 to 0.47], *n* = 53) and Alzheimer’s disease (0.20 [95 % CI: 0.15 to 0.25], *n* = 728). Additional file 2 provides a comprehensive list of results for each condition included in the MEPS model. Additional file 3 lists the same results for NESARC and Additional files 4 and 5 list results for the 12 and 1-month diagnoses in NSMHWB, respecitvely.

The NSMHWB survey asked about one and 12 month diagnoses for a number of mental conditions. To test the sensitivity of this analysis to diagnosis period, we ran the analysis separately for the NSMHWB with one and 12 month diagnoses. Estimated weights for one month diagnoses from the NSMHWB data were higher than those with a 12 month diagnosis. One month prevalence figures were, on average, 18 %, 76 %, 100 %, 13 %, and 32 % higher than 12 month prevalence, for alcohol dependence, anxiety disorders, major depression, dysthymia, and drug dependence, respectively. 12 month diagnosed physical conditions were kept in both analyses and only varied in the range of −5 % to +10 %. The findings indicate that the SF-12 may be quite sensitive to diagnosis periods as it reflects over the longer course of chronic-episodic disorders what the proportion of time without symptoms is. In other words, for longer term prevalence measures, individuals who are not currently symptomatic have a higher probability of being captured. For most chronic conditions in the GBD, estimation approaches are more consistent with the use of 12 month prevalence. For certain conditions, such as major depression which was modeled on the basis of episodes, one month was used [27].

### Model validation

**Fig. 2:**
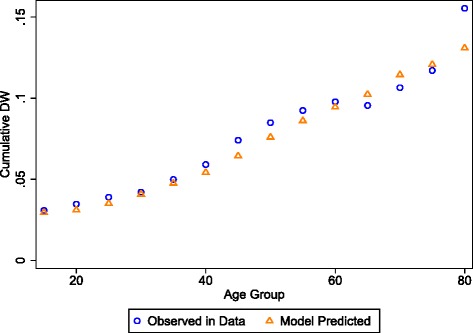
Mean predicted and observed disability weights by age bins. Predictions are carried out using equation 1, where the disability weight given to each condition is that of the mean condition-specific weight as estimated by the model. If the mean weight was negative, the condition weight was truncated to zero, as negative weights are not compatible with the multiplicative comorbidity equation. Each respondent was also given a baseline disability of 0.015, the model estimated intercept

Figure 2 shows the predicted age pattern using our model (equation 1) with the MEPS data against the observed age pattern of disability. After binning the sample into five-year age groups, the model captures the steady rise expected in disability with progression of age. This shows that, at a population level, for any given age, the quantity and composition of conditions alone can explain much of the observed total disability. This highlights the usefulness and necessity of comorbidity adjustment of condition-specific weights when calculating severity distributions, and that the multiplicative model utilized elsewhere in GBD is appropriate for this.

Of note, there is a decline in disability around the age of retirement in the MEPS data that is not reflected by the trend estimated in the comorbid conditions. This improvement in self-reported functional health status around the time of retirement has been described in the literature [28–30], and reflects a subjective limitation of self-report that a condition-only model cannot capture.

### Severity distributions

Table 2 summarizes results of the severity distribution analysis across all three surveys. Full severity distributions were analyzed for 152 conditions. After correcting for comorbid conditions, all causes we analyzed had some proportion of the population in the asymptomatic category. For less severe conditions, such as benign prostatic hyperplasia or alopecia areata, we estimated that 41.5 % [95 % CI: 38.7 %-44.4 %] and 44.1 % [38.7 %-49.4 %] were asymptomatic while for conditions such as chronic ischemic stroke and anxiety disorders 18.6 % [9.5 %-29.9 %] and 28.3 % [26.8 %-29.6 %] of cases were asymptomatic.

**Table 2:** Results of health state severity distribution analysis, as used in the GBD 2013. The cause column represents a GBD cause category, and each row represents a health state, or sequelae, within that cause. When available, distributions from more than one survey were averaged at the draw level. Certain conditions have been included which only have one specified severity category in the GBD DW study but which may have asymptomatic cases. For those conditions, such as benign prostate hyperplasia, the final weight is adjusted for the estimated asymptomatic proportion

			**Combined**			**MEPS**			**AHS 1 month**			**AHS 12 month**			**NESARC**		
**Cause**	**Health State Weight Cutoff**	**DW**	**Mean**	**LCI**	**UCI**	**Mean**	**LCI**	**UCI**	**Mean**	**LCI**	**UCI**	**Mean**	**LCI**	**UCI**	**Mean**	**LCI**	**UCI**
Ischemic heart disease	Asymptomatic	0.000	30.5 %	27.9 %	33.0 %	30.5 %	27.9 %	33.0 %									
Ischemic heart disease	Mild angina due to ischemic heart disease	0.033	24.0 %	16.7 %	30.9 %	24.0 %	16.7 %	30.9 %									
Ischemic heart disease	Moderate angina due to ischemic heart disease	0.080	12.6 %	8.9 %	16.7 %	12.6 %	8.9 %	16.7 %									
Ischemic heart disease	Severe angina due to ischemic heart disease	0.167	33.0 %	27.3 %	39.2 %	33.0 %	27.3 %	39.2 %									
Other cardiovascular and circulatory diseases	Asymptomatic	0.000	18.2 %	15.4 %	21.4 %	18.2 %	15.4 %	21.4 %									
Other cardiovascular and circulatory diseases	Mild heart failure due to other cardiovascular diseases	0.041	55.4 %	48.6 %	60.8 %	55.4 %	48.6 %	60.8 %									
Other cardiovascular and circulatory diseases	Moderate heart failure due to other cardiovascular diseases	0.072	9.9 %	7.0 %	13.5 %	9.9 %	7.0 %	13.5 %									
Other cardiovascular and circulatory diseases	Severe heart failure due to other cardiovascular diseases	0.179	16.5 %	11.7 %	21.2 %	16.5 %	11.7 %	21.2 %									
Other cardiovascular and circulatory diseases	Asymptomatic	0.000	27.5 %	26.6 %	28.5 %	27.5 %	26.6 %	28.5 %									
Other cardiovascular and circulatory diseases	Mild other cardiovascular diseases	0.041	30.2 %	22.8 %	38.6 %	30.2 %	22.8 %	38.6 %									
Other cardiovascular and circulatory diseases	Moderate other cardiovascular diseases	0.072	14.7 %	9.4 %	18.7 %	14.7 %	9.4 %	18.7 %									
Other cardiovascular and circulatory diseases	Severe other cardiovascular diseases	0.179	27.6 %	23.3 %	33.0 %	27.6 %	23.3 %	33.0 %									
Acute hemorrhagic stroke	Asymptomatic	0.000	10.4 %	0.0 %	46.4 %	10.4 %	0.0 %	46.4 %									
Acute hemorrhagic stroke	Acute hemorrhagic stroke severity level 1	0.019	42.1 %	12.5 %	71.4 %	42.1 %	12.5 %	71.4 %									
Acute hemorrhagic stroke	Acute hemorrhagic stroke severity level 2	0.070	22.8 %	0.0 %	53.3 %	22.8 %	0.0 %	53.3 %									
Acute hemorrhagic stroke	Acute hemorrhagic stroke severity level 3	0.316	20.1 %	0.0 %	44.7 %	20.1 %	0.0 %	44.7 %									
Acute hemorrhagic stroke	Acute hemorrhagic stroke severity level 4	0.552	3.9 %	0.0 %	22.2 %	3.9 %	0.0 %	22.2 %									
Acute hemorrhagic stroke	Acute hemorrhagic stroke severity level 5	0.588	0.8 %	0.0 %	11.1 %	0.8 %	0.0 %	11.1 %									
Chronic hemorrhagic stroke	Asymptomatic	0.000	10.4 %	0.0 %	46.4 %	10.4 %	0.0 %	46.4 %									
Chronic hemorrhagic stroke	Chronic hemorrhagic stroke severity level 1	0.019	42.1 %	12.5 %	71.4 %	42.1 %	12.5 %	71.4 %									
Chronic hemorrhagic stroke	Chronic hemorrhagic stroke severity level 2	0.070	22.8 %	0.0 %	53.3 %	22.8 %	0.0 %	53.3 %									
Chronic hemorrhagic stroke	Chronic hemorrhagic stroke severity level 3	0.316	20.1 %	0.0 %	44.7 %	20.1 %	0.0 %	44.7 %									
Chronic hemorrhagic stroke	Chronic hemorrhagic stroke severity level 4	0.552	3.9 %	0.0 %	22.2 %	3.9 %	0.0 %	22.2 %									
Chronic hemorrhagic stroke	Chronic hemorrhagic stroke severity level 5	0.588	0.8 %	0.0 %	11.1 %	0.8 %	0.0 %	11.1 %									
Acute ischemic stroke	Asymptomatic	0.000	18.6 %	9.5 %	29.9 %	18.6 %	9.5 %	29.9 %									
Acute ischemic stroke	Acute ischemic stroke severity level 1	0.019	42.8 %	32.4 %	52.8 %	42.8 %	32.4 %	52.8 %									
Acute ischemic stroke	Acute ischemic stroke severity level 2	0.070	22.7 %	14.4 %	31.5 %	22.7 %	14.4 %	31.5 %									
Acute ischemic stroke	Acute ischemic stroke severity level 3	0.316	11.7 %	5.0 %	19.4 %	11.7 %	5.0 %	19.4 %									
Acute ischemic stroke	Acute ischemic stroke severity level 4	0.552	1.6 %	0.0 %	4.6 %	1.6 %	0.0 %	4.6 %									
Acute ischemic stroke	Acute ischemic stroke severity level 5	0.588	2.5 %	0.7 %	5.4 %	2.5 %	0.7 %	5.4 %									
Chronic ischemic stroke	Asymptomatic	0.000	18.6 %	9.5 %	29.9 %	18.6 %	9.5 %	29.9 %									
Chronic ischemic stroke	Chronic ischemic stroke severity level 1	0.019	42.8 %	32.4 %	52.8 %	42.8 %	32.4 %	52.8 %									
Chronic ischemic stroke	Chronic ischemic stroke severity level 2	0.070	22.7 %	14.4 %	31.5 %	22.7 %	14.4 %	31.5 %									
Chronic ischemic stroke	Chronic ischemic stroke severity level 3	0.316	11.7 %	5.0 %	19.4 %	11.7 %	5.0 %	19.4 %									
Chronic ischemic stroke	Chronic ischemic stroke severity level 4	0.552	1.6 %	0.0 %	4.6 %	1.6 %	0.0 %	4.6 %									
Chronic ischemic stroke	Chronic ischemic stroke severity level 5	0.588	2.5 %	0.7 %	5.4 %	2.5 %	0.7 %	5.4 %									
Endocrine, metabolic, blood, and immune disorders	Asymptomatic	0.000	35.8 %	34.9 %	36.7 %	35.8 %	34.9 %	36.7 %									
Endocrine, metabolic, blood, and immune disorders	Mild endocrine, metabolic, blood, and immune disorders	0.019	42.7 %	36.0 %	47.4 %	42.7 %	36.0 %	47.4 %									
Endocrine, metabolic, blood, and immune disorders	Moderate endocrine, metabolic, blood, and immune disorders	0.145	6.7 %	4.2 %	10.7 %	6.7 %	4.2 %	10.7 %									
Endocrine, metabolic, blood, and immune disorders	Severe endocrine, metabolic, blood, and immune disorders	0.159	14.8 %	12.0 %	17.7 %	14.8 %	12.0 %	17.7 %									
Uterine fibroids	Asymptomatic	0.000	31.5 %	26.0 %	37.4 %	31.5 %	26.0 %	37.4 %									
Other gynecological diseases	Asymptomatic	0.000	34.2 %	33.0 %	35.5 %	34.2 %	33.0 %	35.5 %									
Other gynecological diseases	Mild other gynecological disorders	0.011	45.9 %	38.1 %	52.7 %	45.9 %	38.1 %	52.7 %									
Other gynecological diseases	Moderate other gynecological disorders	0.114	13.2 %	8.0 %	19.6 %	13.2 %	8.0 %	19.6 %									
Other gynecological diseases	Severe other gynecological disorders	0.324	6.6 %	4.6 %	8.9 %	6.6 %	4.6 %	8.9 %									
Alcohol dependence	Asymptomatic	0.000	36.8 %	32.2 %	41.8 %	28.0 %	21.7 %	34.2 %	43.7 %	36.4 %	51.1 %	40.5 %	35.3 %	45.8 %	54.8 %	51.2 %	58.3 %
Alcohol dependence	Very mild alcohol dependence	0.123	53.4 %	49.0 %	58.5 %	51.7 %	43.5 %	59.6 %	46.4 %	39.1 %	54.1 %	50.8 %	45.7 %	56.0 %	40.1 %	36.4 %	43.8 %
Alcohol dependence	Mild alcohol dependence	0.235	3.8 %	2.2 %	5.8 %	5.3 %	2.0 %	9.0 %	3.7 %	1.0 %	7.4 %	3.3 %	1.3 %	5.4 %	2.0 %	1.1 %	2.9 %
Alcohol dependence	Moderate alcohol dependence	0.373	3.4 %	1.6 %	5.3 %	5.8 %	2.4 %	9.6 %	3.4 %	0.9 %	6.9 %	3.0 %	1.4 %	4.8 %	1.6 %	0.8 %	2.6 %
Alcohol dependence	Severe alcohol dependence	0.570	2.6 %	0.9 %	5.7 %	9.2 %	3.6 %	16.3 %	2.7 %	0.5 %	6.3 %	2.4 %	0.5 %	5.5 %	1.5 %	0.5 %	3.0 %
Fetal alcohol syndrome	Asymptomatic	0.000	28.0 %	21.7 %	34.2 %	28.0 %	21.7 %	34.2 %									
Fetal alcohol syndrome	Mild fetal alcohol syndrome	0.016	22.2 %	13.7 %	31.3 %	22.2 %	13.7 %	31.3 %									
Fetal alcohol syndrome	Moderate fetal alcohol syndrome	0.056	24.7 %	17.2 %	32.0 %	24.7 %	17.2 %	32.0 %									
Fetal alcohol syndrome	Severe fetal alcohol syndrome	0.179	25.1 %	17.9 %	33.7 %	25.1 %	17.9 %	33.7 %									
Dysthymia	Asymptomatic	0.000	35.0 %	29.6 %	39.7 %				37.8 %	28.8 %	47.5 %	37.4 %	29.1 %	46.0 %	32.5 %	28.8 %	36.5 %
Major depressive disorder	Asymptomatic	0.000	20.4 %	18.8 %	22.0 %	18.6 %	17.8 %	19.3 %	13.0 %	9.7 %	16.6 %	21.8 %	18.5 %	25.2 %	34.5 %	32.1 %	37.1 %
Major depressive disorder	Mild major depressive disorder	0.145	63.3 %	57.8 %	69.1 %	60.3 %	54.7 %	65.6 %	59.4 %	49.1 %	68.8 %	61.6 %	55.2 %	68.3 %	50.2 %	45.7 %	54.5 %
Major depressive disorder	Moderate major depressive disorder	0.396	9.9 %	8.0 %	11.8 %	11.4 %	9.6 %	13.0 %	17.3 %	12.7 %	22.1 %	10.8 %	7.9 %	13.5 %	7.7 %	6.2 %	9.1 %
Major depressive disorder	Severe major depressive disorder	0.658	6.4 %	3.2 %	11.4 %	9.7 %	5.7 %	15.6 %	10.3 %	3.2 %	19.8 %	5.9 %	1.9 %	11.0 %	7.6 %	4.4 %	12.1 %
Other musculoskeletal disorders	Asymptomatic	0.000	24.9 %	24.3 %	25.4 %	24.9 %	24.3 %	25.4 %									
Other musculoskeletal disorders	Other musculoskeletal disorders severity level 1	0.023	21.5 %	14.5 %	30.4 %	21.5 %	14.5 %	30.4 %									
Other musculoskeletal disorders	Other musculoskeletal disorders severity level 2	0.028	21.3 %	15.7 %	26.9 %	21.3 %	15.7 %	26.9 %									
Other musculoskeletal disorders	Other musculoskeletal disorders severity level 3	0.117	10.9 %	6.5 %	15.8 %	10.9 %	6.5 %	15.8 %									
Other musculoskeletal disorders	Other musculoskeletal disorders severity level 4	0.165	6.2 %	4.6 %	7.8 %	6.2 %	4.6 %	7.8 %									
Other musculoskeletal disorders	Other musculoskeletal disorders severity level 5	0.317	8.0 %	6.4 %	9.4 %	8.0 %	6.4 %	9.4 %									
Other musculoskeletal disorders	Other musculoskeletal disorders severity level 6	0.581	7.3 %	4.1 %	11.9 %	7.3 %	4.1 %	11.9 %									
Low back pain (with leg pain)	Asymptomatic	0.000	21.9 %	20.5 %	23.4 %	21.9 %	20.5 %	23.4 %									
Low back pain (with leg pain)	Mild low back pain with leg pain	0.020	20.9 %	14.5 %	28.6 %	20.9 %	14.5 %	28.6 %									
Low back pain (with leg pain)	Moderate low back pain with leg pain	0.054	28.6 %	22.0 %	34.4 %	28.6 %	22.0 %	34.4 %									
Low back pain (with leg pain)	Severe low back pain with leg pain	0.272	10.5 %	7.8 %	12.6 %	10.5 %	7.8 %	12.6 %									
Low back pain (with leg pain)	Most severe low back pain with leg pain	0.372	18.1 %	11.8 %	25.1 %	18.1 %	11.8 %	25.1 %									
Low back pain (without leg pain)	Asymptomatic	0.000	26.2 %	25.4 %	26.9 %	26.2 %	25.4 %	26.9 %									
Low back pain (without leg pain)	Mild low back pain without leg pain	0.020	28.7 %	21.2 %	37.4 %	28.7 %	21.2 %	37.4 %									
Low back pain (without leg pain)	Moderate low back pain without leg pain	0.054	26.2 %	19.1 %	32.6 %	26.2 %	19.1 %	32.6 %									
Low back pain (without leg pain)	Severe low back pain without leg pain	0.272	7.9 %	6.3 %	9.2 %	7.9 %	6.3 %	9.2 %									
Low back pain (without leg pain)	Most severe low back pain without leg pain	0.372	10.9 %	6.9 %	15.7 %	10.9 %	6.9 %	15.7 %									
Neck pain	Asymptomatic	0.000	33.9 %	31.9 %	36.0 %	33.9 %	31.9 %	36.0 %									
Neck pain	Mild neck pain	0.053	45.4 %	38.3 %	50.7 %	45.4 %	38.3 %	50.7 %									
Neck pain	Moderate neck pain	0.114	7.5 %	4.2 %	12.2 %	7.5 %	4.2 %	12.2 %									
Neck pain	Severe neck pain	0.229	4.0 %	2.5 %	5.4 %	4.0 %	2.5 %	5.4 %									
Neck pain	Most severe neck pain	0.304	9.2 %	6.1 %	13.0 %	9.2 %	6.1 %	13.0 %									
Other oral disorders	Asymptomatic	0.000	34.2 %	32.5 %	35.8 %	34.2 %	32.5 %	35.8 %									
Other oral disorders	Mild other oral disorders	0.006	29.4 %	22.7 %	36.5 %	29.4 %	22.7 %	36.5 %									
Other oral disorders	Severe other oral disorders	0.051	36.5 %	29.4 %	43.4 %	36.5 %	29.4 %	43.4 %									
Asthma	Asymptomatic	0.000	35.9 %	34.3 %	37.5 %	30.3 %	29.2 %	31.3 %	38.9 %	35.9 %	41.9 %	38.5 %	35.3 %	42.1 %			
Asthma	Controlled asthma	0.015	23.2 %	15.8 %	32.3 %	20.5 %	13.7 %	28.6 %	24.1 %	15.8 %	33.6 %	25.0 %	17.1 %	34.9 %			
Asthma	Partially controlled asthma	0.036	21.5 %	15.0 %	27.7 %	22.3 %	16.6 %	28.1 %	21.5 %	14.1 %	28.1 %	20.8 %	13.8 %	27.4 %			
Asthma	Uncontrolled asthma	0.133	19.4 %	15.1 %	26.5 %	26.9 %	21.4 %	35.1 %	15.5 %	11.1 %	22.5 %	15.7 %	11.5 %	22.2 %			
Interstitial lung disease and pulmonary sarcoidosis	Asymptomatic	0.000	19.7 %	12.3 %	27.5 %	19.7 %	12.3 %	27.5 %									
Interstitial lung disease and pulmonary sarcoidosis	Mild interstitial lung disease and pulmonary sarcoidosis	0.019	45.4 %	35.4 %	55.1 %	45.4 %	35.4 %	55.1 %									
Interstitial lung disease and pulmonary sarcoidosis	Moderate interstitial lung disease and pulmonary sarcoidosis	0.225	13.3 %	6.1 %	21.9 %	13.3 %	6.1 %	21.9 %									
Interstitial lung disease and pulmonary sarcoidosis	Severe interstitial lung disease and pulmonary sarcoidosis including heart failure	0.408	21.6 %	11.5 %	32.7 %	21.6 %	11.5 %	32.7 %									
Other pneumoconiosis	Asymptomatic	0.000	22.4 %	13.3 %	31.6 %	22.4 %	13.3 %	31.6 %									
Other pneumoconiosis	Mild other pneumoconiosis	0.019	32.0 %	20.7 %	42.9 %	32.0 %	20.7 %	42.9 %									
Other pneumoconiosis	Moderate other pneumoconiosis	0.225	12.7 %	4.7 %	23.8 %	12.7 %	4.7 %	23.8 %									
Other pneumoconiosis	Severe other pneumoconiosis including heart failure	0.408	32.9 %	19.0 %	47.5 %	32.9 %	19.0 %	47.5 %									
Other sense organ diseases	Asymptomatic	0.000	38.9 %	37.7 %	40.1 %	38.9 %	37.7 %	40.1 %									
Other sense organ diseases	Mild other sense organ diseases	0.006	11.5 %	6.3 %	19.1 %	11.5 %	6.3 %	19.1 %									
Other sense organ diseases	Moderate other sense organ diseases	0.011	28.4 %	21.9 %	34.6 %	28.4 %	21.9 %	34.6 %									
Other sense organ diseases	Severe other sense organ diseases	0.113	21.3 %	15.5 %	28.5 %	21.3 %	15.5 %	28.5 %									
Alopecia areata	Asymptomatic	0.000	44.1 %	38.7 %	49.4 %	44.1 %	38.7 %	49.4 %									
Alopecia areata	Mild alopecia areata	0.011	31.9 %	23.3 %	39.7 %	31.9 %	23.3 %	39.7 %									
Alopecia areata	Severe alopecia areata	0.067	24.0 %	15.5 %	32.5 %	24.0 %	15.5 %	32.5 %									
Decubitus ulcer	Asymptomatic	0.000	26.3 %	23.8 %	28.8 %	26.3 %	23.8 %	28.8 %									
Decubitus ulcer	Mild decubitus ulcer	0.011	19.9 %	13.9 %	26.2 %	19.9 %	13.9 %	26.2 %									
Decubitus ulcer	Moderate decubitus ulcer	0.067	28.9 %	22.8 %	34.8 %	28.9 %	22.8 %	34.8 %									
Decubitus ulcer	Severe decubitus ulcer	0.405	24.9 %	18.8 %	30.7 %	24.9 %	18.8 %	30.7 %									
Dermatitis	Asymptomatic	0.000	41.8 %	39.9 %	43.8 %	41.8 %	39.9 %	43.8 %									
Dermatitis	Mild contact dermatitis	0.027	50.1 %	45.5 %	53.2 %	50.1 %	45.5 %	53.2 %									
Dermatitis	Severe contact dermatitis	0.188	8.0 %	5.7 %	12.2 %	8.0 %	5.7 %	12.2 %									
Eczema	Asymptomatic	0.000	41.8 %	39.9 %	43.8 %	41.8 %	39.9 %	43.8 %									
Eczema	Mild eczema	0.027	50.1 %	45.5 %	53.2 %	50.1 %	45.5 %	53.2 %									
Eczema	Moderate eczema	0.188	5.7 %	3.8 %	8.6 %	5.7 %	3.8 %	8.6 %									
Eczema	Severe eczema	0.576	2.4 %	1.4 %	4.1 %	2.4 %	1.4 %	4.1 %									
Cellulitis	Asymptomatic	0.000	33.2 %	31.2 %	35.4 %	33.2 %	31.2 %	35.4 %									
Cellulitis	Mild cellulitis	0.027	46.7 %	40.8 %	51.0 %	46.7 %	40.8 %	51.0 %									
Cellulitis	Severe cellulitis	0.188	20.1 %	15.7 %	25.8 %	20.1 %	15.7 %	25.8 %									
Other skin and subcutaneous diseases	Asymptomatic	0.000	44.6 %	43.2 %	45.9 %	44.6 %	43.2 %	45.9 %									
Psoriasis	Asymptomatic	0.000	38.3 %	34.6 %	42.1 %	38.3 %	34.6 %	42.1 %									
Psoriasis	Mild psoriasis	0.027	50.0 %	44.3 %	54.7 %	50.0 %	44.3 %	54.7 %									
Psoriasis	Moderate psoriasis	0.188	6.8 %	4.5 %	10.6 %	6.8 %	4.5 %	10.6 %									
Psoriasis	Severe psoriasis	0.576	4.8 %	3.0 %	7.1 %	4.8 %	3.0 %	7.1 %									
Urticaria	Asymptomatic	0.000	38.1 %	33.0 %	43.4 %	38.1 %	33.0 %	43.4 %									
Urticaria	Mild urticaria	0.027	47.8 %	40.3 %	53.9 %	47.8 %	40.3 %	53.9 %									
Urticaria	Severe urticaria	0.188	14.2 %	9.8 %	20.8 %	14.2 %	9.8 %	20.8 %									
Viral warts	Asymptomatic	0.000	44.1 %	40.8 %	47.5 %	44.1 %	40.8 %	47.5 %									
Viral warts	Mild viral warts	0.006	32.1 %	25.6 %	39.4 %	32.1 %	25.6 %	39.4 %									
Viral warts	Severe viral warts	0.067	23.8 %	17.1 %	29.8 %	23.8 %	17.1 %	29.8 %									
Molluscum contagiosum	Asymptomatic	0.000	44.1 %	40.8 %	47.5 %	44.1 %	40.8 %	47.5 %									
Molluscum contagiosum	Mild molluscum contagiosum	0.006	32.1 %	25.6 %	39.4 %	32.1 %	25.6 %	39.4 %									
Molluscum contagiosum	Severe molluscum contagiosum	0.067	23.8 %	17.1 %	29.8 %	23.8 %	17.1 %	29.8 %									
Benign prostatic hyperplasia	Asymptomatic	0.000	41.5 %	38.7 %	44.4 %	41.5 %	38.7 %	44.4 %									
Interstitial nephritis and urinary tract infections	Asymptomatic	0.000	33.1 %	31.4 %	35.0 %	33.1 %	31.4 %	35.0 %									
Interstitial nephritis and urinary tract infections	Mild interstitial nephritis and urinary tract infections	0.006	23.5 %	16.3 %	30.7 %	23.5 %	16.3 %	30.7 %									
Interstitial nephritis and urinary tract infections	Moderate interstitial nephritis and urinary tract infections	0.051	43.4 %	35.9 %	50.4 %	43.4 %	35.9 %	50.4 %									
Amphetamine use disorders	Asymptomatic	0.000	54.8 %	39.6 %	71.4 %										54.8 %	39.6 %	71.4 %
Amphetamine use disorders	Mild amphetamine dependence	0.079	38.6 %	23.1 %	54.5 %										38.6 %	23.2 %	54.5 %
Amphetamine use disorders	Severe amphetamine dependence	0.486	6.5 %	1.0 %	13.8 %										6.5 %	1.0 %	13.8 %
Cannabis use disorders	Asymptomatic	0.000	57.9 %	51.4 %	63.3 %										57.9 %	51.5 %	63.3 %
Cannabis use disorders	Mild cannabis dependence	0.039	36.1 %	30.6 %	42.3 %										36.1 %	30.6 %	42.3 %
Cannabis use disorders	Severe cannabis dependence	0.266	6.0 %	3.6 %	8.4 %										6.0 %	3.6 %	8.4 %
Cocaine use disorders	Asymptomatic	0.000	50.4 %	36.5 %	63.8 %										50.4 %	36.6 %	63.7 %
Cocaine use disorders	Mild cocaine dependence	0.116	42.8 %	29.7 %	57.9 %										42.8 %	29.8 %	57.7 %
Cocaine use disorders	Severe cocaine dependence	0.479	6.8 %	1.7 %	13.2 %										6.8 %	1.8 %	13.1 %
Opioid use disorders	Asymptomatic	0.000	52.2 %	41.3 %	62.3 %										52.2 %	41.4 %	62.2 %
Opioid use disorders	Mild opioid dependence	0.335	42.0 %	30.8 %	53.6 %										42.0 %	30.8 %	53.5 %
Opioid use disorders	Severe opioid dependence	0.697	5.8 %	0.8 %	12.7 %										5.8 %	0.8 %	12.6 %
Anxiety disorders	Asymptomatic	0.000	28.3 %	26.8 %	29.6 %	25.0 %	24.3 %	25.9 %	18.2 %	14.7 %	21.5 %	22.1 %	19.1 %	25.1 %	47.8 %	45.9 %	50.1 %
Anxiety disorders	Mild anxiety disorders	0.030	40.9 %	33.0 %	47.2 %	44.3 %	35.9 %	50.1 %	41.4 %	32.4 %	50.0 %	43.9 %	34.0 %	51.5 %	34.1 %	29.0 %	38.0 %
Anxiety disorders	Moderate anxiety disorders	0.133	18.5 %	13.8 %	23.8 %	18.2 %	13.6 %	24.0 %	24.4 %	17.8 %	31.2 %	21.5 %	16.2 %	28.6 %	9.9 %	7.3 %	12.7 %
Anxiety disorders	Severe anxiety disorders	0.523	12.3 %	8.2 %	17.4 %	12.5 %	8.9 %	17.3 %	16.0 %	9.6 %	23.1 %	12.5 %	7.5 %	18.4 %	8.3 %	5.6 %	11.2 %

The asympotmatic category represents not only the percentage of individuals with disease and no symptoms but, given the random timing of the survey relative to health fluctuations, can also capture the fluctuation in and out of symptoms over time in the population with the condition. For example, it is not possible for individuals who are never symptomatic to be diagnosed with anxiety, but rather that those individuals are not symptomatic all the time. In other words, some proportion of individuals with diagnosed anxiety in the past year would not be symptomatic at the time of the survey.

**Fig. 3:**
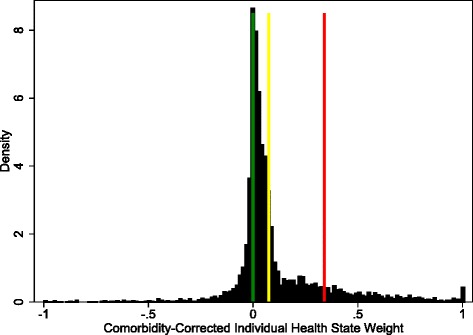
Histogram of estimated health state disability weights for anxiety cases in a MEPS sample. Lines represent cutoffs in severity, moving from asymptomatic (green), to severe (red). The dark area in each bin represents the proportion of the population with each comorbidity-correct health state; in this case 25 % asymptomatic, 45 % mild, 18 % moderate and 13 % severe

Of the 37 conditions analyzed and used in GBD 2013, four conditions (dysthymia, other skin conditions, benign prostatic hyperplasia, and uterine fybroids) had only one GBD health state weight, so the final average disability weight was simply taken as that health state weight times the proportion symptomatic. However, most conditions included in this analysis do have several defined health states. For example, anxiety disorders are valuated as mild (mean health state weight = 0.03), moderate (0.13), and severe (0.52). This analysis estimated the average population proportions in these states to be 40.9 %, 18.5 %, and 12.3 % respectively, leaving 28.3 % asymptomatic. A histogram of comorbidity-corrected anxiety weights from MEPS is provided in Fig. 3 to illustrate how this is done. Anxiety is commonly comorbid with depression and a number of other mental health and substance use disorders [31], meaning that we would expect the comorbidity correction to push the distribution downward. Despite the removal of comorbidities, a large number of individuals remained on the higher end of the distribution, the interpretation being that they had a high anxiety-attributable disability.

One should not assess the severity of any condition relative to another based on the proportion asymptomatic alone. Utlimately, these distributions are used to take a weighted mean of all disability weights associated with that condition. These severity-adjusted disability weights are mutliplied by prevalence to estimate YLDs. Thus, the final severity-adjusted weight of a health state also depends on the severity distribution of the symptomatic proportion relative to DW cutoffs from the GBD study.

The distributions in Table 2 represent the outputs in the of the analysis as described in the methods section of this paper which were incorporated into the GBD 2013 round of estimates. It should be noted that for some causes GBD analysts and collaborating groups may have added further analytical methods to come to their results as appropriate to their specific modelling strategies. For example, the group estimating the burden of low back pain did not include an asymptomatic category in their analysis, as it did not correspond to their particular estimation strategy using point prevalence [32].

We were able to draw from more than one of the data sources for nine conditions. For most others except the drug use disorder categories we used MEPS only. In most cases, there was a high level of agreement among the distributions estimated from the different surveys. For any condition that was tracked in more than one survey, the final distribution was taken as a mean across all estimated distributions at the draw level in order to incorportate uncertainty from all sources.

## DISCUSSION

The severity distributions estimated here from three surveys suggest that a substantial fraction of individuals who report a condition or meet diagnostic criteria for a condition such as alcohol use or anxiety disorders show no demonstrable increase in functional limitation once comorbidities have been taken into account. For some this may be surprising but it confirms the underlying premise motivating this study: that there is substantial heterogeneity in severity of outcomes which should be accounted for in making population health estimates. In applying these empirical findings to DALY estimates as part of the GBD 2013 Study, we move closer to more accurately describing the non-fatal burden of many diseases. The finding further highlights the need to collect more detailed information on severity in groups of individuals with various conditions that fully captures the range of other comorbidities that may be present and may be key determinants of the level of reported health functioning.

A simple multiplicative model of comorbidities explains much of the observed age pattern of functional health limitations. In other words, comorbid conditions, especially when an extensive list is used such as in MEPS, provide a reasonable accounting of individual functional impairments. A mapping from SF-12 to disability weights allows for this analysis to serve as a direct input into non-fatal outcome estimation in the GBD. Given the importance for the GBD of assessing marginal severity distributions for conditions, this opens up the possibility of more extensive use of functional health status information in future efforts at quantifying the burden of disease.

The purpose of this analysis was to distribute cases into coarse severity bins as defined by the disability weight study in order to create final severity-weighted disability weights for conditions whose multiple levels of severity were already built into the study. It should be noted that final weighted disability weights are quite sensitive to the cutoffs used for the bins. In future analyses, with access to more data, researchers should consider using the full range of severity reported for each condition rather than binning into a few categories. Severity distributions could thus be independent of predefined health state weights.

This study has several key limitations. First, the mapping from SF-12 MCS and PCS values into the GBD disability weight space was based on a few small convenience samples covering only 62 conditions. All respondents completing these SF-12 responses for the hypothetical health status lived in Seattle or attended a GBD workshop in Greece. There may be cultural variation in the way different individuals may map a lay description into an SF-12 score which is not explored or captured in this analysis. Second, this study uses data from only two countries, the US and Australia. Generalization of societal values across geography and populations of different social economic status in applying disability weights has been a topic of debate [33–35]. Moreover, access to health services is higher in these two countries compared to many other countries in the world. Applying the severity distributions from these two countries to DALY estimates for all world regions means that we are unable to capture a worse severity distribution in populations that lack access to health care interventions that ameliorate symptoms and improve functioning. Unfortunately, these are the only large samples with both multiple conditions and SF-12 data that we have been able to identify. In the systematic reviews on the severity distribution for major disabling diseases the vast majority of data also come from high-income regions with good access to care and the few data points from low- or middle-income countries are often biased also towards people who are under care. Therefore, the lack of differentiation in severity by access to care is not just a problem in this analysis but a more general data source weakness in GBD estimates of non-fatal disease.

This study is further limited by its reliance on the SF-12 summary measures to bear the weight of a rather complicated analysis covering a broad spectrum of conditions. The assumption implicit here is that the PCS and MCS dimensions capture all health impacts due to conditions present in the regression. While countless studies have demonstrated the SF-12’s usefulness as a tool in measuring health status for a variety of physical and mental conditions, it is hard to imagine that it can fully capture within its limited dimensions, and with a high degree of sensitivity, the different types of health loss caused by all conditions that we tracked. It is possible that some conditions could be biased to zero if their symptoms did not contribute to the health dimensions captured in SF-12. SF-12 could further potentially bias the results of some conditions if some related important driving comorbidity was not included; for example if depression was not included, anxiety would look much worse, though we believe this concern is addressed by the large number of conditions tracked, particularly in the MEPS dataset.

Care should be taken when interpreting results for particular conditions. For example, we chose not to include schizophrenia in this analysis as household surveys exclude institutionalized and homeless populations and may further exclude people with schizophrenia differentially by non-response [36]. Hence, for schizophrenia, GBD analysts chose to rely on pooled estimates of severity from the epidemiological literature rather than the results from this analysis [27]. Similarly, household surveys tend to underestimate the true prevalence of drug dependence, but particularly so for opioid dependence, in which case GBD analysts for that condition applied an empircal correction factor [26]. It is up to individual researchers to undertand the data, methods, and limitations when applying results of analyses such as this to their causes of interest.

There is great potential in national burden of disease studies of using multi-round functional health status information to more precisely and comparably measure the severity distributions of important conditions in different settings. For GBD, replication of such data collections in low- and middle-income countries would be highly desirable as a complement to this analysis of surveys from two high-income countries which we had access to. A key design factor for such studies in countries with less access to health care would be to select an unbiased sample from the population rather than those who are receiving care. Such studies should also include anchoring vignettes or other strategies designed to adjust for possible differential item functioning [16].

Existing survey data such as MEPS, NESARC, or NSMHWB that have collected SF-12 data and information on the presence of a series of comorbid conditions can be used to fill critical gaps in the information on condition severity. The results provide an empirical basis for assessing the marginal distribution of severity controlling for comorbidity which is required for the GBD. The systematic reviews conducted for GBD found that existing information on severity distribution is scarce or not harmonious for the majority of disabling chronic conditions contributing to global YLDs. The analysis of these three surveys has provided new insight into key aspects of making comparable measurements of severity across a broad range of conditions. Measurement of the severity of any condition is influenced by co-existing conditions that have similar symptoms (such as pain, restricted mobility, or mental health symptoms). Ignoring comorbidity leads to overestimation of severity particularly in conditions that are most common in the elderly or for mental disorders where comorbidity with another mental or substance use disorder is common. Additionally, there is always a proportion of cases which report no disability that can be ascribed to the condition after correcting for disability from comorbid conditions. For some conditions, this is more likely due to fluctuation of symptoms over the course of a disorder rather than reflecting a sub-set of people with the condition who do not experience any disability at all. Ignoring this fluctuation in symptoms as many studies measuring severity do (rarely is an asymptomatic category explicitly measured) leads to an overestimation of the severity distribution.

## Additional files

Additional file 1:
**Lay descriptions and their disability weights, and the estimated composite SF-12 scores for each health state.** The full SF-12 to Disability Weight mapping function can be replicated in R using the loess command with a span of 0.88 on the data in this table. (XLSX 13 kb) Additional file 2:
**MEPS Model Results.** (XLSX 18 kb) Additional file 3:
**NESARC Model Results.** (XLSX 10 kb) Additional file 4:
**NSMHWB 12 month Model Results.** (XLSX 10 kb) Additional file 5:
**NSMHWB 1 month Model Results**. (XLSX 10 kb) Additional file 6:
**Mapping from ICD-9 to GBD causes.** (XLSX 15 kb)

## Abbreviations

COPD: Chronic obstructive pulmonary disease
DALY: Disease-adjusted life year
DSM-IV: Diagnostic and statistical manual of mental disorders, 4^th^ edition
GBD: Global burden of disease, injuries, and risk factors study
ICD: International classification of disease revision
MCS: Mental component score (of SF-12)
MEPS: Medical expenditure panel survey
NESARC: National epidemiological survey on alcohol related condition
NSMHWB: Australian national survey of mental health and wellbeing of adults
PCS: Physical component score (of SF-12)
SF-12: The 12-item short form questionnaire
YLD: Years lived with disability
YLL: Years of life lost
